# A novel deleterious *PTEN* mutation in a patient with early-onset bilateral breast cancer

**DOI:** 10.1186/1471-2407-14-70

**Published:** 2014-02-06

**Authors:** Laura Maria Pradella, Cecilia Evangelisti, Claudia Ligorio, Claudio Ceccarelli, Iria Neri, Roberta Zuntini, Laura Benedetta Amato, Simona Ferrari, Alberto Maria Martelli, Giuseppe Gasparre, Daniela Turchetti

**Affiliations:** 1 Department of Medical and Surgical Sciences, Unit of Medical Genetics, University of Bologna, Via Massarenti 9, 40138 Bologna, Italy; 2 Department of Biomedical and Neuromotor Sciences, University of Bologna, Bologna, Italy; 3 Section of Anatomic Pathology “M. Malpighi”, University of Bologna, Bellaria Hospital, Bologna, Italy; 4 Department of Experimental, Diagnostic and Specialty Medicine, Unit of Pathology, University of Bologna, Bologna, Italy; 5 Department of Experimental, Diagnostic and Specialty Medicine, Unit of Dermatology, University of Bologna, Bologna, Italy

**Keywords:** Hereditary breast cancer, PTEN, Cowden syndrome, PI3K/Akt/mTOR pathway

## Abstract

**Background:**

An early age at Breast Cancer (BC) onset may be a hallmark of inherited predisposition, but BRCA1/2 mutations are only found in a minority of younger BC patients. Among the others, a fraction may carry mutations in rarer BC genes, such as *TP53*, *STK11*, *CDH1* and *PTEN.* As the identification of women harboring such mutations allows for targeted risk-management, the knowledge of associated manifestations and an accurate clinical and family history evaluation are warranted.

**Case presentation:**

We describe the case of a woman who developed an infiltrating ductal carcinoma of the right breast at the age of 32, a contralateral BC at age 36 and another BC of the right breast at 40. When she was 39 years-old, during a dermatological examination, mucocutaneous features suggestive of Cowden Syndrome, a disorder associated to germ-line *PTEN* mutations, were noticed. *PTEN* genetic testing revealed the novel c.71A > T (p.Asp24Val) mutation, whose deleterious effect, suggested by conservation data and *in silico* tools, was definitely demonstrated by the incapacity of mutant PTEN to inhibit Akt phosphorylation when used to complement PTEN-null cells. In BC tissue, despite the absence of LOH or somatic mutations of *PTEN*, Akt phosphorylation was markedly increased in comparison to normal tissue, thus implying additional somatic events into the deregulation of the PI3K/Akt/mTOR pathway and, presumably, into carcinogenesis. Hence, known oncogenic mutations in *PIK3CA* (exons 10 and 21) and *AKT1* (exon 2) were screened in tumor DNA with negative results, which suggests that the responsible somatic event(s) is a different, uncommon one.

**Conclusion:**

This case stresses the importance of clinical/genetic assessment of early-onset BC patients in order to identify mutation carriers, who are at high risk of new events, so requiring tailored management. Moreover, it revealed a novel *PTEN* mutation with pathogenic effect, pointing out, however, the need for further efforts to elucidate the molecular steps of *PTEN-*associated carcinogenesis.

## Background

A young age at Breast Cancer (BC) onset may be a hallmark feature of inherited predisposition. Indeed, germ-line mutations in the two major BC genes BRCA1 and BRCA2 have been reported in 15 to 23% of younger Italian BC patients [1,2], consistently with frequencies described in other western populations, which range from 6 to 23% [3-5].

Although the majority of the remaining cases might be explained by a multifactorial etiology, mutations in rarer BC predisposing genes should also be considered. Rare, high penetrance BC genes include *TP53*, *STK11*, *CDH1* and *PTEN*. Collectively, they are generally thought to account for less than 1% of inherited BC; nevertheless, an accurate clinical and family history evaluation may provide significant clues to the identification of patients carrying such uncommon mutations. Recognizing mutation carriers is crucial to plan targeted risk-management according to the specific gene, as is routinely done for BRCA mutation carriers.

The *PTEN* gene encodes a negative regulator of the PI3K/Akt/mTOR pathway and is one of the most frequently mutated genes in cancer, with loss of heterozygosity at the *PTEN* locus being reported in about 40% of invasive BC [6,7]. Conversely, germ-line *PTEN* mutations are rare, and cause several syndromes with variable clinical manifestations that are collectively labeled *PTEN Hamartoma Tumor Syndrome* (PHTS). The prototypic syndrome, Cowden Syndrome (CS), is featured by macrocephaly, gastrointestinal lesions and cerebellar gangliocytoma, as well as by benign and malignant tumors of the thyroid, the endometrium and the breast, with a lifetime risk of BC estimated to be as high as 85.2% [8]. Nevertheless, the most typical features are specific mucocutaneous lesions, which include trichilemmomas, acral keratoses and oral papillomatous papules, and occur in 90-100% of cases.

Here we describe a patient with early-onset, metachronous bilateral BC, who presented with mucocutaneous features suggesting CS and was found to carry a novel missense *PTEN* mutation. Functional assessment of the mutation allowed to demonstrate it is associated to loss of function.

## Case presentation

### Clinical history

An Italian woman underwent lumpectomy of the right breast with axillary dissection at the age of 32 years for Invasive Ductal Carcinoma at stage pT2N1a(1/35)M0. The tumor was ER and PR positive (both 90%) and HER2/neu negative. She received postoperative radiotherapy, chemotherapy (Epirubicin plus CMF) and Tamoxifen. Four years later, at the age of 36, she was diagnosed with contralateral breast cancer and total left mastectomy was performed. Pathologic examination revealed a Invasive Ductal Carcinoma (0.12 cm in diameter) in a context of Ductal Carcinoma *In Situ* of cribriform and micropapillary type. Sentinel lymph node was negative; ER and PR expression positive (90% and 40%, respectively), HER2/neu negative. She was prescribed endocrine treatment with LHRH agonist and anastrozole.

**Figure 1:**
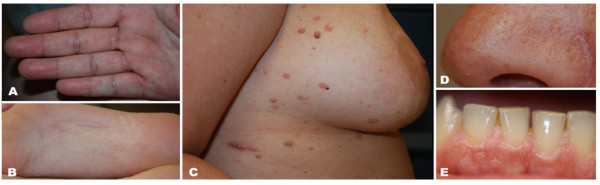
**Cutaneous findings of our case. A** and **B)** Palmoplantar keratosis; **C)** Multiple seborrheic Keratoses and benign lymphangiomatosis papules lesions that occur as asymptomatic erythematous grouped vesicles overlying the radiation field on the right chest. **D)** Small papules on the nose **E)** Multiple small papules on the gingiva with cobblestone appearance.

When she was 39 years-old, during a dermatological examination, mucocutaneous features suggestive of CS were noticed (Figure 1). The patient was therefore referred to the genetic clinic: family history was unremarkable, whereas previous clinical manifestations in the patient were also consistent with CS; indeed, at the age of 28 she underwent thyroidectomy for goiter and at 31 she had a uterine leyomioma removed; she also had several skin lesions removed, such as lymphangiomata of the trunk, lipomas, keratoses. In addition, clinical examination revealed macrocephaly (head circumference: 61 cm). The diagnosis of CS was therefore confirmed according to the criteria of the National Comprehensive Cancer Network (http://www.nccn.org), and proper surveillance recommended [8].

At the age of 40, another tumor was detected at the right breast, consisting of a multicentric Invasive Ductal Carcinoma rypT1c(m) with negative ER/PR and positive HER2/neu (80%). After total right mastectomy was performed, adjuvant chemotherapy with Paclitaxel and Trastuzumab was undertaken, which, however, was discontinued after 3 months due to the occurrence of interstitial pneumonia. Pulmonary function was promptly recovered after treatment discontinuation and steroids administration, and the patient is currently disease-free after 30 months.

### Gene testing

The mutational analysis of *PTEN* was performed by sequencing all 9 exons of the gene, as well as the splice-junctions and the gene promoter region, in DNA extracted from peripheral lymphocytes of the patient, after informed consent was collected and blood sample obtained.

**Figure 2:**
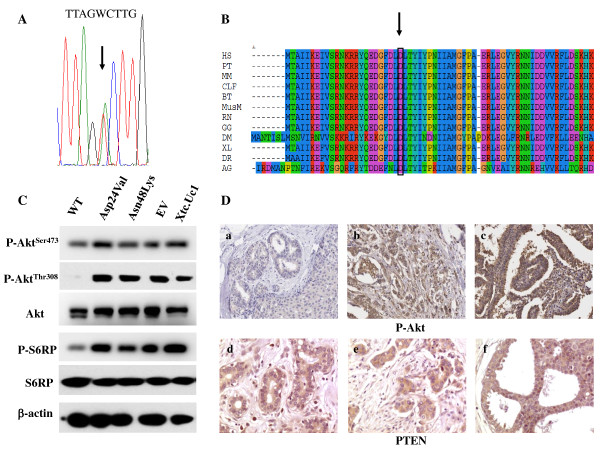
**Genetic and molecular characterization of PTEN c.71A>T mutation. A)** Electropherogram showing PTEN c.71A > T transversion; **B)** PTEN amino acidic sequence alignment among species: *H.sapiens* [HS], *P.troglodytes* [PT], *M.mulatta* [MM], *C.lupus* [CLP], *B.taurus* [BT], *M.musculus* [MusM], *R.norvegicus* [RN], *G.gallus* [GG], *X.laevis* [XL], *D.rerio* [DR], *D.melanogaster* [DM] and *A.gambiae* [AG]; **C)** Western Blot analysis on total cell lysates of XTC.UC1 cells tranfected with wild type PTEN [WT], PTEN^Asp24Val^, PTEN^Asn48Lys^, on Empty Vector [EV], and on untransfected XTC.UC1 cells, showing that only PTEN WT is able to inhibit P-Akt^Ser473^, P-Akt^Thr308^ and P-S6RP; **D)** Immunohistochemical analyses of the breast tumors [Magnification 200X]: Phospho-AKT-Ser473 immunostaining, showing negative reaction in normal breast duct **(a)**, strong nuclear and cytoplasmic immunoreactivity in infiltrating **(b)** and *in situ***(c)** breast tumors; PTEN immunostaining, showing normal reaction in normal duct **(d)** and in breast infiltrating and *in situ* carcinomas **(e, f)**.

The analysis revealed the heterozygous transversion c.71A > T in exon 1 (RefSeq NG_007466), predicting the substitution of a residue of aspartic acid with a valine at codon 24 (p.Asp24Val) (Figure 2A). To our knowledge, this mutation had not been reported before; in addition, it was absent in the database of pathogenic mutations Human Gene Mutation Database-HGMD (http://www.hgmd.cf.ac.uk/ac/index.php/) and was neither reported in the Exome Variant Server (http://evs.gs.washington.edu/EVS/), nor in the 1000 genomes browser (http://browser.1000genomes.org/index.html). Furthermore, 96 controls from the same geographical area of the patient were screened for the mutation, without finding any carrier. The analysis performed on PTEN homologous aminoacid sequences downloaded from HomoloGene (http://www.ncbi.nlm.nih.gov/homologene) and multialigned with MUSCLE (http://www.ebi.ac.uk/Tools/msa/muscle/) showed that Asp24 is highly conserved among metazoans (Figure 2B); accordingly, the *in silico* tools SIFT and Polyphen2 predicted a deleterious effect of the variant, with scores of 0 and 0.942, respectively.

### Functional assessment of the mutation

*PTEN* full length cDNA was cloned in a pCDNA 3.1(−) (Invitrogen, Life Technologies Ltd, UK) expression vector and site-directed mutagenesis was performed in order to obtain the mutation under study (PTEN^Asp24Val^) and the known pathogenic mutation Asn48Lys (PTEN^Asn48Lys^) [9]. These mutant forms, as well as the wild-type PTEN, were used to complement XTC.UC1 cells; XTC.UC1 is a cell line established from a metastasis of thyroid oncocytic follicular carcinoma [10], which we found to be null for PTEN (Additional file 1: Figure S1).

PTEN specific Western Blot analysis confirmed that both the wild-type and the mutated proteins were expressed; hence, their ability to regulate the PI3K/Akt/mTOR pathway was investigated by assessing the relative amount of the phosphorylated forms of AKT (at Ser473 and Thr308) and of the mTORC1 substrate 40S ribosomal protein S6 (S6RP), in comparison to the respective total counterparts. Only wild-type PTEN was found to inhibit AKT and S6RP phosphorylation in complemented cells, whereas PTEN^Asp24Val^ appeared, conversely, to lose PTEN lipid phosphatase function, analogously to the PTEN^Asn48Lys^ (Figure 2C). Thus, the p.Asp24Val mutation was proven to be deleterious.

### PTEN and AKT immunostaining

Immunostaining was performed on paraffin-embedded, formalin-fixed tissue of the first two breast tumors (right IDC and left DCIS). PTEN was normally expressed in both, with a sub-cellular distribution similar to the corresponding normal breast tissue; conversely, p-AKT (Ser473) staining showed a markedly increased AKT phosphorylation in the tumors, compared to the normal tissue (Figure 2D), suggesting that the PI3K/Akt/mTOR pathway is over-activated in the tumor, unlike in normal tissue.

### Screening of somatic mutations in *PTEN, PI3KCA* and *AKT*

Analysis of the *PTEN* coding sequence in the DNA extracted from the *in situ* breast carcinoma showed the c.71A > T to be heterozygous, thus ruling out the loss of the wild-type allele, and failed to detect any additional sequence variants. Recurrent oncogenic mutations in exon 10 and 21 of *PI3KCA* and exon 2 of *AKT,* which are known to cause activation of the PI3K/Akt/mTOR pathway in several cancer types, were also excluded by sequence analysis.

## Conclusions

Women harboring a predisposing gene mutation face a high risk to develop BC at a young age and to experience multiple primary BCs. These risks have been extensively studied in women with BRCA1/2 mutations, whose average age at BC onset is around 45 years, while the risk of contralateral BC 25 years after first BC is 47.4%, according to recent figures [11].

For rarer conditions, such as CS, these risks and the relative management are not so clearly established. Until recently, lifetime BC risk in CS female patients was largely accepted to be around 25-50%; however, recent reports on two large series raised the estimated lifetime risk to 77-85% [8,12]. Consistently, a pooled analysis of literature data and records from the Mayo Clinic provided an estimate of the cumulative BC risk as high as 81% [13]. The age at BC onset is generally believed to be young (38–50 years) [14]. Nevertheless, data on the risk for bilateral BC are scarce; in the French series, out of 23 BC cases, 11 (48%) were reported as bilateral [12], while in the study by Riegert-Johnson and colleagues 34% of patients diagnosed with BC cancer had bilateral disease [13].

The case here reported adds evidence to the hypothesis that *PTEN* mutation carriers are at high risk for early-onset and multiple BCs, thus pointing out the need to promptly identify these women in order to properly manage such risks, similarly to BRCA carriers. To this aim, it is crucial that clinicians caring for BC patients are aware of associated manifestations suggesting rare genetic syndromes, and/or that every patient with early-onset BC is referred to cancer genetic assessment.

Missense variants of uncertain significance are a relatively common finding that complicates the interpretation of gene test results. In our patient, we found the novel missense mutation p.Asp24Val, which had not been reported before. Anyway, different mutations at the same codon had been described: the germ-line p.Asp24Tyr mutation, in a Bannayan-Riley-Ruvalcaba patient [15], and the p.Asp24Gly, detected in the germline of a CS patient [16] as well as in sporadic tumors of the endometrium and of the central nervous system as a somatic mutation (COSM5170, http://www.sanger.ac.uk/genetics/CGP/cosmic/), supporting the functional relevance of the highly conserved Asp24. These data, together with the *in silico* prediction, strongly suggested a functional role of this variant, which was confirmed by demonstrating *in vitro* that the mutant PTEN, unlike the wild-type protein, was unable to inhibit the PI3K/Akt/mTOR pathway.

Whether *PTEN* acts as a classical tumor suppressor gene following Knudson’s two-hit hypothesis is still controversial: on one hand, in a mouse model of prostate cancer, the complete loss of PTEN, unlike its dose-reduction, was demonstrated to induce senescence instead of cancer, unless the loss of TP53 co-occurred [17]. On the other hand, PTEN immunostaining was proven to be negative in 13 out of 15 BC samples from CS patients, suggesting loss or inactivation of the wild-type *PTEN* allele in the tumor [18]. To explain its heterogeneous behavior, *PTEN* has been recently appointed as a haploinsufficient gene, characterized, however, by tissue specificity and context dependency [19].

In the present case, the mutation was demonstrated to be at the heterozygous state in both normal and tumor tissue and no additional *PTEN* sequence mutations were detected in the tumor, which is in line with what we found in non-breast tumors from other CS patients, where loss or mutations of the wild-type *PTEN* allele were excluded. In such cases, however, somatic mutations of different genes were found, with an apparent correlation between the specific gene and the type of tumor developed [20,21]. In the present case, the markedly increased Akt phosphorylation detected in BC, if compared to normal tissue, led us to infer that a somatic event had cooperated in deregulating the PI3K/Akt/mTOR pathway and, presumably, in inducing carcinogenesis. Hence, activating mutations commonly found to impair PI3K/Akt/mTOR regulation in cancer were analyzed in the breast tumor, with none of them being detected. Such findings suggest the involvement of a different, uncommon somatic event, such as the possible epigenetic inactivation of the wild-type *PTEN* allele (which could not be ruled out) or an unusual mutation in genes involved in the PI3K/Akt/mTOR pathway, thus demonstrating once again how carcinogenesis in *PTEN* mutation carriers is a complex, still elusive process, which requires major efforts to be elucidated.

### Ethics appoval and patient consent

Clinical assessment and genetic testing in this patient were performed primarily for diagnostic purposes. Genetic counselling and testing were carried out in agreement with the European guidelines. The patient gave her informed consent to diagnostic genetic testing and to additional analyses needed to obtain insights on the significance of the mutation found. The consent form for genetictests that she signed had been previously approved by the Ethical and Legal Board of the Policlinico S.Orsola-Malpighi. Written informed consent was obtained from the patient for the publication of this case report and any accompanying images. A copy of the written consent is available for review by the Editor of this journal.

## Competing interests

The authors declare that they have no competing interests.

## Authors’ contributions

LMP designed and interpreted PTEN mutational analysis, performed PTEN cloning, carried out molecular analyses in tumour tissue and contributed to draft the manuscript. CE performed and interpreted WB analysis on PI3K/Akt/mTORpathway. CL and CC performed and interpreted immunohistochemical analyses of P-AKT and PTEN, respectively. IN carried out dermatological examinations. RZ participated in cloning. LA performed constitutional PTEN gene testing. SF participated in conceiving and interpreting molecular analyses. AMM coordinated and interpreted WB studies on the PI3K/Akt/mTOR pathway. GG coordinated and interpreted molecular studies and participated in drafting the manuscript. DT provided genetic counselling and performed clinical examinations in the index case, designed and coordinated the studies and drafted the manuscript. All authors read and approved the final manuscript.

## Pre-publication history

The pre-publication history for this paper can be accessed here:

http://www.biomedcentral.com/1471-2407/14/70/prepub

## Supplementary Material

Additional file 1: Figure S1 PTEN-null status of XTC.UC1 cells. (A) Electropherogram showing the hemizygous c.210delT within exon 4 of the *PTEN* gene in XTC.UC1 cells compared to a wild-type control (lower panel). The wild-type c.210delT is underlined. (B) Western blot for PTEN confirming lack of the full-length protein in XTC.UC1 cells compared to a control. Beta-actin was used as a loading control. Click here for file
