# Supplementary material for: A novel deleterious PTEN mutation in a patient with early-onset bilateral breast cancer
Source: BMC Cancer. 2014 Feb 6;14:70. doi: 10.1186/1471-2407-14-70 (PMC3922036; doi:10.1186/1471-2407-14-70)
Supplement: Additional file 1: Figure S1 — PTEN-null status of XTC.UC1 cells. (A) Electropherogram showing the hemizygous c.210delT within exon 4 of the PTEN gene in XTC.UC1 cells compared to a wild-type control (lower panel). The wild-type c.210delT is underlined. (B) Western blot for PTEN confirming lack of the full-length protein in XTC.UC1 cells compared to a control. Beta-actin was used as a loading control. [file 1471-2407-14-70-S1.ppt]

## Slide 1
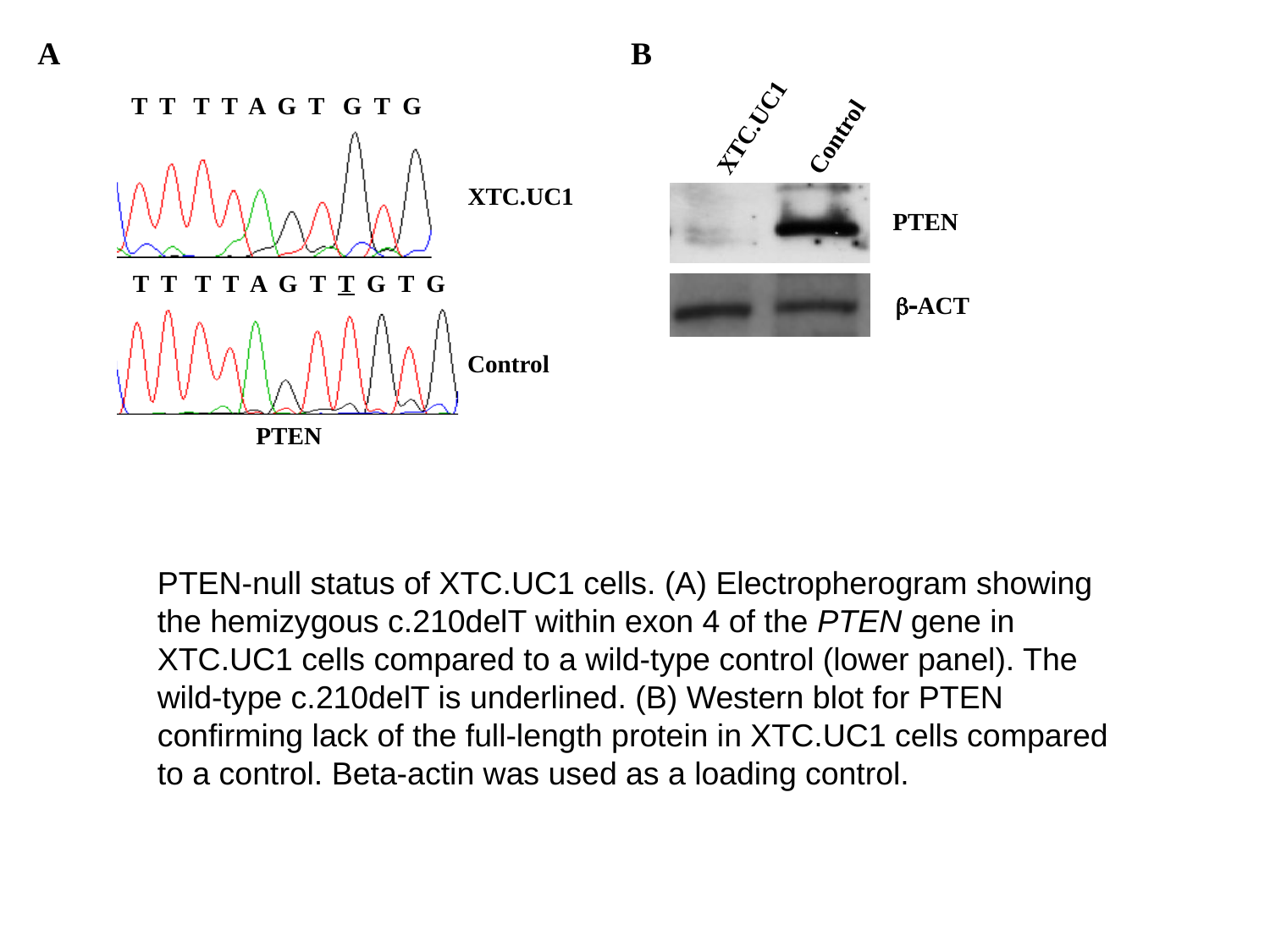

A
B
T T T T A G T G T G
XTC.UC1
T T T T A G T T G T G
Control
PTEN
XTC.UC1
Control
PTEN
β−ACT
PTEN-null status of XTC.UC1 cells. (A) Electropherogram showing the hemizygous c.210delT within exon 4 of the PTEN gene in XTC.UC1 cells compared to a wild-type control (lower panel). The wild-type c.210delT is underlined. (B) Western blot for PTEN confirming lack of the full-length protein in XTC.UC1 cells compared to a control. Beta-actin was used as a loading control.
